# Primary ovarian leiomyoma: a rare ovarian tumour

**DOI:** 10.1007/s00404-022-06842-4

**Published:** 2022-12-21

**Authors:** Mascha Pervan, Michael Gembicki, Henriette Princk, Achim Rody, Lars Hanker, Franziska Hemptenmacher, Maggie Banys-Paluchowski

**Affiliations:** Department of Gynaecology and Obstetrics, University Hospital Schleswig-Holstein, Campus Lubeck, Lubeck, Germany

**Keywords:** Myoma, Ovarian tumour, Leiomyoma, Laparoscopy

## Abstract

After performing laparoscopic unilateral adnexectomy in a 53-year-old woman for a rapidly grown unilateral adnexal mass, pathologists reported a primary ovarian leiomyoma with no genuine ovarian tissue. This rare diagnosis is found in less than 100 reports after systematic literature review, a greater number of asymptomatic ovarian leiomyomas can be expected. Thorough preoperative diagnostic measures are essential as rare cases of malignancy have been described.

## What does this study add to the clinical work

**Table:** 

Leiomyomas typically appear in the uterus but can also form in rare other locations as shown in this case, so this highlights the importance to consider a myoma as a diffenrential diagnosis for tumours of uncertain dignity.	

## Presentation

**Fig. 1:**
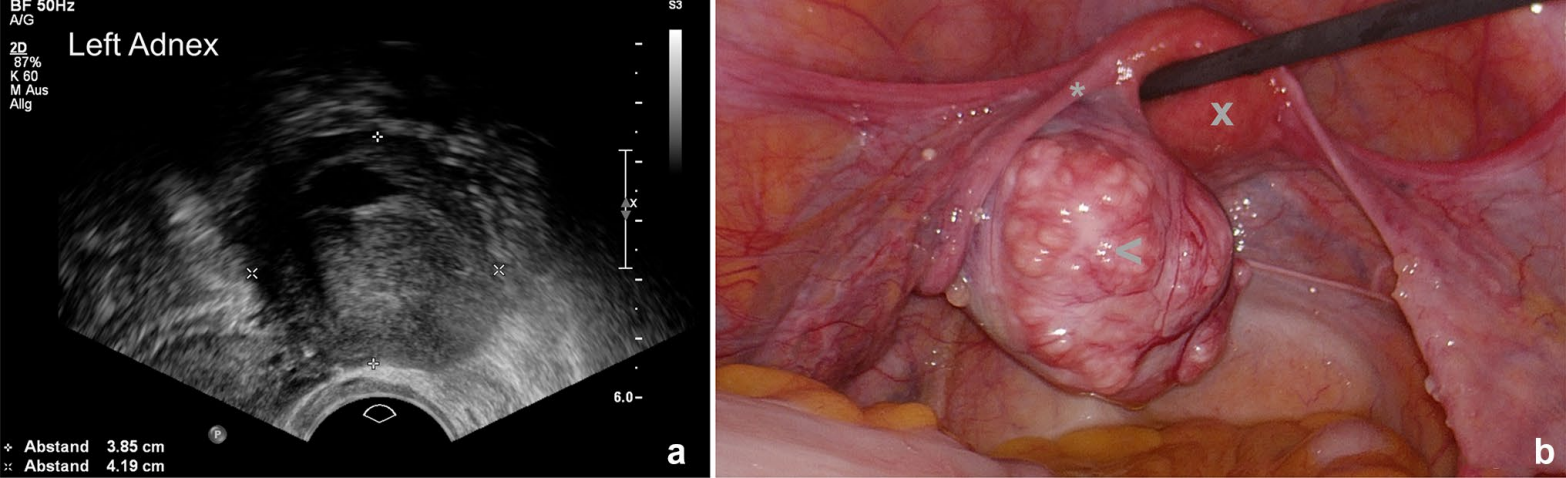
**a** IOTA-Sonogram of left adnex, **b** Laparoscopic image of the primary ovarian leiomyoma (<) with adjacent left fallopian tube (*) and uterus (x)

A 53-year-old postmenopausal woman was referred to our tertiary referral university hospital with a rapidly grown unilateral adnexal mass and abnormal sonogram. She experienced no specific symptoms or discomfort, CA-125 level was normal. We carried out IOTA ADNEX model-sonography and estimated a 95% probability for benignity with suspected ovarian fibroma (Fig. 1a). Laparoscopic unilateral adnexectomy was performed. The ovary presented with an uneven but smooth surface with increased vascularity (Fig. 1b). Postoperative recovery was normal. The pathology report described a primary ovarian leiomyoma with no genuine ovarian tissue.

Discussion: systematic literature review revealed less than 100 reports of primary ovarian leiomyomas, with some cases finding residual ovarian tissue [1, 2]. Up to 85% of cases are found in premenopausal women [3]. Presentation mainly occurs due to symptoms like unilateral lower abdominal pain with palpable mass or menstrual disorders. Sonograms usually show large tumours measuring 5–15 cm with normal CA-125. Smooth muscle cells of the hilum vessels are discussed to be the origin [2], a greater number of undiagnosed small, asymptomatic ovarian leiomyomas can be expected. Thorough preoperative diagnostic measures are essential as cases of malignant primary ovarian leiomyosarcoma (POLMS) have been described [4].

## Data Availability

Published data can be provided at the author's discretion.
